# Metataxonomic and Histopathological Study of Rabbit Epizootic Enteropathy in Mexico

**DOI:** 10.3390/ani10060936

**Published:** 2020-05-28

**Authors:** Xiao-Haitzi Daniel Puón-Peláez, Neil Ross McEwan, José Guadalupe Gómez-Soto, Roberto Carlos Álvarez-Martínez, Andrea Margarita Olvera-Ramírez

**Affiliations:** 1 Doctorado en Ciencias Biológicas, Facultad de Ciencias Naturales, Universidad Autónoma de Querétaro, Avenida de las Ciencias S/N Juriquilla, Delegación Santa Rosa Jáuregui, Santiago de Querétaro, Qro. C.P. 76230, Mexico; xh.puon@gmail.com; 2 School of Pharmacy & Life Sciences, Robert Gordon University, Garthdee Road, Aberdeen AB10 7GJ, UK; n.mcewan@rgu.ac.uk; 3 Cuerpo Académico de Nutrición y Reproducción Animal, Facultad de Ciencias Naturales, Universidad Autónoma de Querétaro, Avenida de las Ciencias S/N Juriquilla, Delegación Santa Rosa Jáuregui, Santiago de Querétaro, Qro. C.P. 76230, Mexico; jose.gomez@uaq.mx; 4 Licenciatura en Microbiología, Facultad de Ciencias Naturales, Universidad Autónoma de Querétaro, Av. Junipero Serra, Antiguo Aeropuerto, Campus Aeropuerto S/N. Santiago de Querétaro, Qro. C.P. 76140, Mexico; roberto.alvarez@uaq.mx; 5 Cuerpo Académico Salud Animal y Microbiología Ambiental, Facultad de Ciencias Naturales, Universidad Autónoma de Querétaro, Avenida de las Ciencias S/N Juriquilla, Delegación Santa Rosa Jáuregui, Santiago de Querétaro C.P. 76230, Mexico

**Keywords:** rabbits, ERE, etiology

## Abstract

**Simple Summary:**

Epizootic rabbit enteropathy (ERE) is a worldwide-distributed dysbiotic syndrome that affects young rabbits. In Mexico, ERE represents 32% of the enteropathies that occur in rabbit production farms. The etiology of this syndrome has not been clarified yet; however, it has been associated with nutritional, environmental, and microbial factors. A metataxonomic and histopathology study of ERE was carried out to compare the lesions and gastrointestinal microbiota of healthy and positive-ERE rabbits. The results revealed a difference in the diversity and abundance of the gastrointestinal microbiota in rabbits with ERE. The genus *Clostridium* and the species. *Cloacibacillus porcorum* and *Akkermansia muciniphila* were associated with the presentation of ERE. Histopathologic analysis showed smaller crypt sizes in the colon of ERE rabbits.

**Abstract:**

Epizootic rabbit enteropathy (ERE) affects young rabbits and represents 32% of the enteropathies in rabbit production farms in Mexico. The etiology of this syndrome has not been clarified yet. A metataxonomic and histopathology study of ERE was carried out to compare the gastrointestinal microbiota and histopathological lesions of healthy and positive-ERE rabbits. The metataxonomic study was done using an Illumina MiSeq (MiSeq^®^ system, Illumina, San Diego California, USA) massive segmentation platform, and a Divisive Amplicon Denoising Algorithm 2 (DADA2 algorithm) was used to obtain Shannon and Simpson diversity indices as well as the relative abundance of the identified communities. For the histopathological study, paraffin sections of the cecum, ileo-cecal valve, and colon were stained with eosin and hematoxylin. AxioVision 4.9 software (Carl Zeiss MicroImaging GmbH, Jena, Germany) was used to measure the crypt depths. Statistical analysis was done using PERMANOVA analysis for the metataxonomic study and ANOVA for the histopathology study. Histopathologic analysis showed smaller sizes of crypts in the colon of ERE rabbits. Differences were observed in the diversity and abundance of the gastrointestinal microbiota between the analyzed groups. The genus *Clostridium* and the species *Cloacibacillus porcorum* and *Akkermansia muciniphila* were associated with ERE. The results obtained from this study can provide information for future clarification of the etiology and proposals of effective treatments.

## 1. Introduction

Rabbit epizootic enteropathy (ERE) is a digestive syndrome, which has had a negative impact on rabbit production since the 1990s [1,2]. ERE can affect up to 95% of animals in any production system and it can reach levels of approximately 90% morbidity and 80% mortality [3]. Rabbits between 3 and 7 weeks are highly susceptible to ERE and reduce their daily food consumption for approximately 2 weeks, which causes a decrease in growth and daily weight gain [3,4].

In Mexico, ERE was first observed towards the end of 2001 or the beginning of 2002, affecting different production centers, but mainly in rabbits between 5 and 7 weeks of age [5]. An incidence of abundance of 32% has been reported with ERE [6], presenting highly variable mortality rates ranging from 30% to 70% [7]. Non-specialized feeding in semi-intensive systems in rabbit production increases the risk of developing ERE in Mexico. Additionally, the microbial profile of the digestive tract of the female rabbit determines the profile of her litter [8], meaning, if the mother has a dysbiosis in her microbial profile, the entire litter will be affected.

ERE can be difficult to diagnose due to the similarity of the signs between this and other enteropathies [9]. During ERE outbreaks, rabbits reduce food and water intake, and in extreme cases, they will stop eating and drinking. Affected rabbits can show mild diarrhea and translucent mucus [7,10]. However, the most common signs are abdomen bloating, generalized dilatation in the gastrointestinal tract, cecal paralysis in some cases, and the presence of abundant mucus [11].

After necropsy, animals can show a distended stomach and small intestine with the presence of gaseous and aqueous contents. In the cecum, there is translucent and prominent mucus [10,12,13]. ERE is characterized by an absence of any inflammatory or congestive lesions; however, goblet cell hyperplasia has been described in the small intestine [4,9,14]. In addition, certain chemical alterations lead to changes in the pH of the ileum and colon [10]. There is a decrease in the pH of the stomach, as well as in part of the duodenum and urine. This decrease in pH is believed to be due to the lack of food in the stomach, while the increase in pH in the colon is due to microbial dysbiosis [4,15]. 

The first metataxonomic studies on ERE reported changes in the abundances of certain microorganisms, such as: *Clostridium* spp., *Bacteroides* spp., *Ruminococcus* spp. [16], *Akkermansia muciniphila*, Bacteroides-Prevotella, *Clostridium coccoides*, *Methanobrevi bacter* [15], *Bacteroides, Blautia, Dorea, Clostridia*, and a number of unclassified organisms [17]. These studies used the second-generation platform 454 pyrosequencing. Recently, Jin et al. [18] used a second-generation platform Illumina, MiSeq system. They reported a change in six genera (*Bacteroides, Rikenella, Akkermansia*, *Escherichia, Lysinibacillus*, and *Clostridium*), meaning organisms from five families (Bacteroidaceae, Verrucomicrobiaceae, Enterobacteriaceae, Planococcaceae, and Clostridiaceae) and three phyla (Bacteroidetes, Verrucomicrobia, and Firmicutes) were affected. They also reported the presence of the phylum Sinergetes, mainly the genus *Cloacibacillus porcorum*, which is related to the gastrointestinal microbiome in pigs [19] and oral problems (periodontitis) in bovines [20].

In the present work, a histopathological study and metataxonomic analysis was performed using a second-generation Illumina MiSeq sequencing [21] of the V3–V4 region of the ribosomal 16s gene to get the microbial profile of healthy and ERE rabbits of an intensive rabbit farm in Mexico.

## 2. Materials and Methods 

The study was approved by the Bioethics Committee of the Faculty of Natural Sciences of the Autonomous University of Querétaro (number 93FCN2016).

The study was undertaken in a commercial meat rabbit production farm localized 19°45′ north latitude and 101°03′ west longitude, at an altitude of 1900 m above sea level (Jaripeo, Charo municipality, Morelia Michoacán, Mexico). This farm typically has 100 breeding animals (80 females and 20 males) and animals for meat production are housed at a typical density of 7 animals per 0.54 m^2^. Typically, in total, there are normally 660 animals housed in a single room (100 breeders and 560 for meat). Animals were fed with a standard commercial diet (crude protein: 16%, crude fiber: 17%) and had access to water ad libitum. The diet contained no anticoccodic or antibiotic compounds. All rabbits were weaned at 35 days old. Six rabbits aged 42 days with ERE (mean weight: 449.16 g) were chosen; ERE diagnosis was determined based on clinical signs, such as diarrhea, distended abdomen, mucus excretion, cecal impaction, decrease of food intake, and bruxism [7,11]. In addition, six healthy rabbits (mean weight of 483.5 g) were chosen from the same feeding and handling group. Selected rabbits were placed in individual cages (60 × 90 × 40 cm) in 2 groups: healthy and ERE positive. The groups were housed in the quarantine zone of the same farm in Morelia. 

Euthanasia was performed during the same day for all animals according to NOM-062-ZOO-1999 [22], by cervical dislocation through mechanical traction and jugular exsanguination. The healthy group was euthanized and sampled first in the trail area of the farm. Subsequently, the facilities and materials were cleaned and disinfected. The ERE group was then euthanized using the same protocol. 

Immediately after being euthanized, an incision was made into the abdomen to expose the stomach, small intestine, cecum, and large intestine. A hemp thread ligation was performed in the final portion of the small intestine and another at the end of the ileo-ceco-colic valve. Subsequently, 2 ligatures were made for the cut, one 0.5 cm before the ligation of the small intestine and another 0.5 cm after the ligation of the ileo-ceco-colic valve, and the cut was performed between both ligatures.

To obtain intestinal tissue, a ligation was performed at the beginning of the ileo-ceco-colic valve, another at 10 cm after the ligation of the small intestine (beginning of the colon), and a further 3 cm from the previous ligation. Cuts were made to obtain the area in the ligated ileo-ceco-colic valve and colon, which were cleaned with phosphate buffered solution (PBS) and then infiltrated with 10% PBS buffered formalin (pH 7) and immersed in flasks with the same formalin composition.

In the case of the cecum, a longitudinal cut was made and washed with PBS, then it was held in place with pins on cork circles of 2 cm diameter and immersed in bottles with buffered 10% formalin (pH 7). In this way, samples from the cecum, ileum, and colon were obtained for histopathology analysis. The bottles were stored at room temperature and transferred to the Animal Nutrition Laboratory, Natural Sciences Faculty of the Autonomous University of Queretaro (FCN, UAQ) for further analysis. Furthermore, the entire contents of the cecum were poured into a 15-mL RNase and DNase free plastic tube, followed by releasing the ligation of the small intestine. Once the tube was filled, this was immediately frozen in a −20 °C freezer for storage and transport to the Laboratory Veterinary Microbiology, FCN, UAQ, where samples were stored at −80 °C for further analysis.

For histopathological analysis, the tissue samples were washed with saline and embedded in paraffin, then 5-µm sections were cut from each sample. Staining was carried out using the hematoxylin and eosin technique. Stained slides were analyzed by light microscopy (Vert. A1, CarlZeiss Microscopy, Gottingen, Germany). Crypt depth was measured in the crypts when there was a complete longitudinal section, a villus, and its associated crypt. The depth of the crypt was measured from the crypt–villus junction to the base using AxioVision 4.9 software (Carl Zeiss MicroImaging GmbH, Jena, Germany). In total, 10 crypts were measured for each sample [23]. A completely randomized design and a Tukey test [24] with SAS 2008 software (Statistical Analysis System, SAS Institute, Cary, North Carolina, USA) were used for comparison between samples.

For metataxonomic analysis, the cecal content of each sample was freeze-dried as follows: 1.5 mL of cecal content of each sample was placed in a 2-mL tube free of DNases and RNAses. Tubes were covered with Parafilm and some holes were made in the Parafilm with a sterile needle. The tube was placed into a flask (Fast-Freeze ™ Flasks, Labconco ™, Kansas MO, USA). The flask was chilled for 24 h at 0 °C. The refrigerated flask was placed in the freeze dryer (FreeZone ™ Freeze-Dry Systems 4.5 L, Labconco ™, Kansas MO, USA) for 48 h of drying.

DNA extraction of lyophilized cecal samples was performed with a QIAamp DNA Stool Mini Kit (Qiagen, Venlo, The Netherlands) following the manufacturer’s instructions as follows. Disruption of cells was performed using 200 mg of silica beads (0.1 mm) placed in a 2-mL tube free of DNases and RNAses. Afterwards, 50 mg of lyophilized cecal sample and 1.4 mL of stool lysis buffer (ASL) were added. Homogenization with a vortex was performed for 1 min and the sample was heated for 5 min at 95 °C, homogenized by vortexing for 15 s, and centrifuged for 1 min at 14,000× *g*. Then, 1.2 mL of sample was transferred to a new 2-mL tube and 1 InhibitEX tablet was added. Homogenization was performed for 1 min and incubated at room temperature for 1 min. Centrifugation was performed for 5 min at 14,000× *g*. The supernatant was placed in a new 1.5-mL tube along with 15 µL of proteinase K, 200 µL supernatant, and 200 µL of Lysis Buffer (AL) solution, which was mixed for 15 s. This was incubated for 10 min at 70 °C. After this, purification and elution were performed as described in the manufacturer’s instructions, and DNA was eluted was stored at −20 °C.

A standardization of the DNA concentration of each sample was performed to give a concentration of 50 ng/µL, and sequencing of the DNA of the cecal contents was performed with the second-generation DNA sequencing platform (MiSeq^®^ system, Illumina, San Diego California, USA), where the V3–V4 region of the 16S rRNA gene was used (Zymo Research Corp Irvine California USA). Sequencing was performed using genomic DNA, i.e., without a prior round of polymerase chain reaction (PCR) before sequencing. The MiSeq™ sequencing used a v3 reagent kit with 600 cycles and >10% PhiX spike-in and 2 × 250 bp chemistry.

The protocol after sequencing was as follows: Filtering, identifying unique sequences (Dereplication), constructing tables of operational taxonomic units, removing chimeric sequences, assigning taxonomic identification, and determining abundance and diversity.

Sequences were processed by filtering the low-quality reads and trimming the sequencing adapters, which was determined with the quality score function, and trim and filter function of the DADA2 package, which also removed chimeric sequences. This cut-off was used to achieve a maximum of 2 errors per read and a size of 540 in reads forward and 150 in reads reverse, leaving approximately 480bp readings with Trimmomatic software Bolger [25], and an inspection of the data quality was made with FastQC (Babraham Bioinformatics, Babraham, Cambridge, UK), and again later using the R Project software for Statistical Computing following the work guide proposed by Callahan et al. [21]. The following packages were used: Divisive Amplicon Denoising Algorithm 2 (DADA2), DECIPHER (DECIPHER Project Manage, Cambrige, UK), Phyloseq Microbiome, Vegan, and Ggplot2 (R packages, Wellignton New Zealand). Taxonomy assignment was performed using Uclust from Qiime v.1.9.1 (Quantitative Insights Into Microbial Ecology, Colorado, USA) and compared against the internally designed and curated Zymo 16S Research Database (Zymo Research California, USA). Composition visualization, alpha-diversity, and beta-diversity analyses were performed with Qiime v.1.9.1. For the comparison of the sequences identified by DADA2, a PERMANOVA test was performed following the methodology described in the Vegan package manual [26]. A rarefaction curve was produced with the rarefy script: Rarefaction Species Richness in Vegan: Community Ecology Package [26]. In the case of the analysis of bacterial diversity, this was evaluated by calculating the Shannon index, the Simpson index, the Chao1 index and an estimate of coverage based on abundance (ACE), following the methodology proposed in the Vegan package manual [26].

The operational taxonomic units (OTUs) and abundance tables were used for the linear discriminant analysis effect size (LEfSe) calculation. The metadata information compared (ERE vs. healthy) was used. Subsequently, the biomarkers of each class and visualization of the representative OTUs were generated.

## 3. Results

### 3.1. Histopathological Study

**Figure 1:**
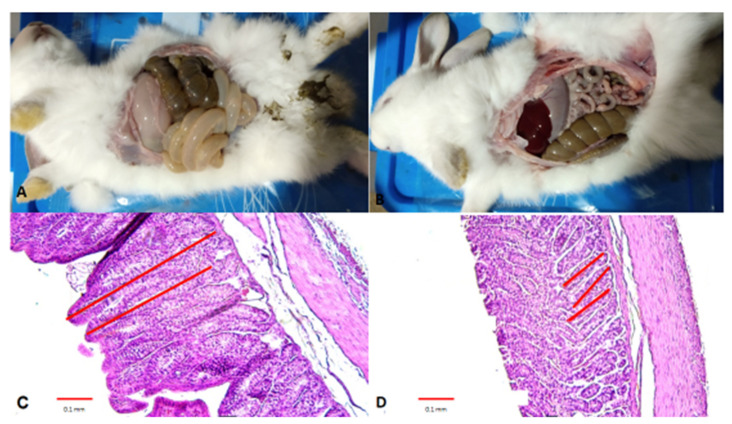
Histopathological study of rabbit digestive tracts. (**A**) Image of necropsy of a healthy rabbit; (**B**) Image of necropsy of an Epizootic rabbit enteropathy (ERE)-infected rabbit; (**C**) Section of villi of the colon of a healthy rabbit; (**D**) Section of villi from an ERE-infected rabbit colon. For (**C**,**D**), the red lines show the length of the crypts.

The findings at necropsy were consistent in all infected rabbits with ERE. The stomach was full of liquid, without the presence of food, and slightly ischemic. They also presented with an accumulation of liquid, mucus, and gas in the small intestine and colon, causing bowel distension and loss of visibility of the intestinal austras. The cecum had an accumulation of liquid and gas, as well as food; in two cases the food was impacted, and the liquid was scarce. In the case of healthy rabbits, no pathological change was observed (Figure 1A,B).

The histological sections of ERE rabbits were a smaller size of the crypts in the colon section (Figure 1C,D). In healthy sections, no change was reported in the crypts (Table 1). Other histological changes were not observed in the epithelium.

### 3.2. Metataxonomic Study

#### 3.2.1. Bacterial Abundance

**Figure 2:**
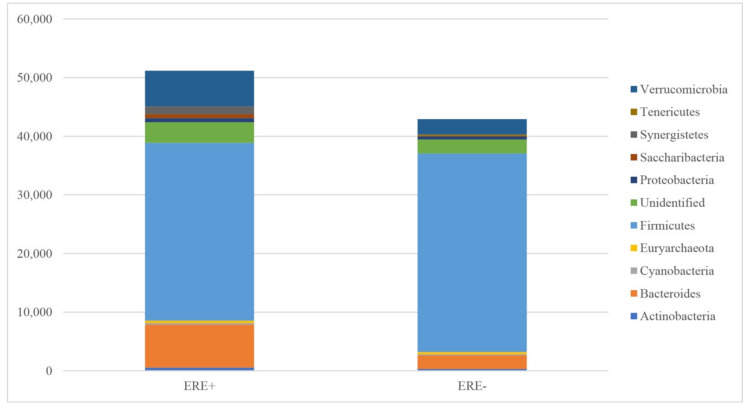
The abundance of bacterial genera in cecal samples of healthy and ERE rabbits. ERE+: Rabbits positive for epizootic enteropathy. ERE−: Healthy rabbits.

**Figure 3:**
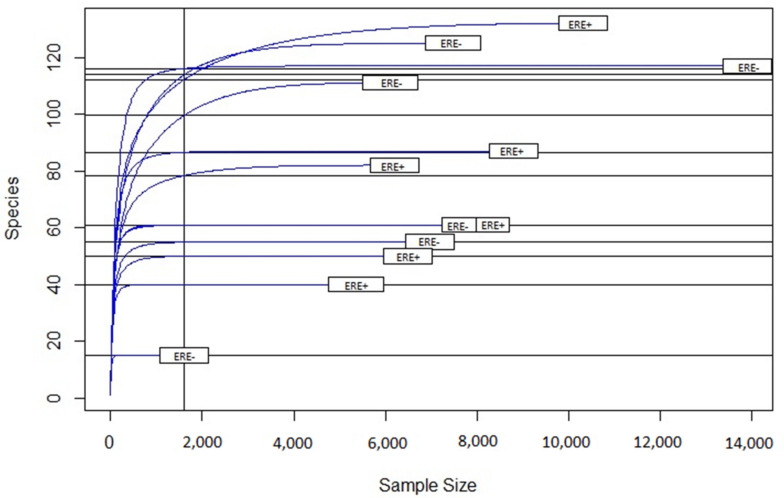
Rarefaction curve for sequence analysis. The vertical axis shows the number of different species (Operational taxonomic units, OTUs) that would be expected to be found after sampling the number of sequences shown on the horizontal axis. The curvature towards the horizontal indicates the greater sequencing effort required to observe new species. The curve was obtained with the Rarefaction Species Richness script from the Vegan package on the Rstudio platform [27].

**Figure 4:**
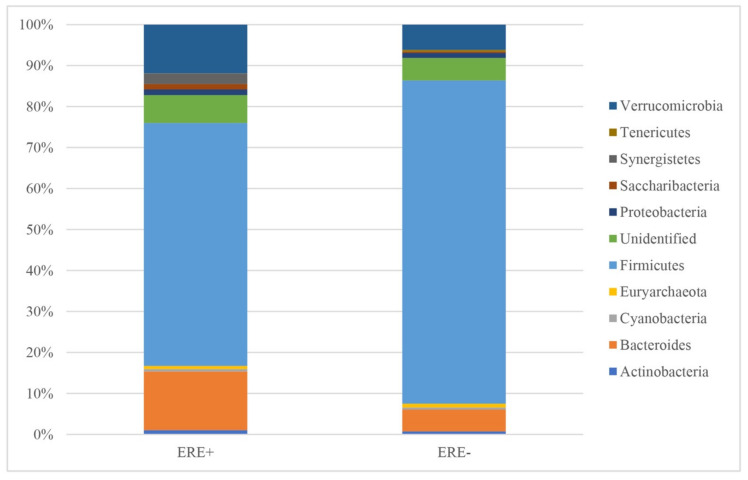
The relative abundance of bacterial genera in cecal samples of healthy and ERE rabbits. ERE+: Rabbits positive for epizootic enteropathy. ERE−: Healthy rabbits.

The samples from the ERE-positive rabbits yielded more sequences relative to those from healthy animals (Figure 2). Following rarefaction curve analysis, an almost horizontal asymptotic curve was obtained for almost all individuals (Figure 3). This indicates that the number of species (based on the number of OTUs) was no longer increasing in relation to the number of sequenced amplicons included in the species identification. In addition, at the level of individual animals, there were more OTUs per rabbit in healthy animals than there were in those that had previously had the disease. Full details of the respective abundance of each Phylum, Class, Order, Family and Genus are shown in the Supplementary Table S1 In the ERE-positive rabbits, the most abundant phyla were: Firmicutes, Bacteroides, and Verrucomicrobia. The least abundant phyla detected were Tenericutes, Cyanobacteria, and Euryachaeota. In healthy rabbits, the most abundant phyla were Firmicutes, Verrucomicrobia, and Bacteroides, with Saccharibacteria, Cyanobacteria, and Tenericutes as the least abundant detectable phyla. The phylum Synergistetes was only seen in rabbits with ERE. In addition, there were a number of unidentified sequences (mean of 3505 for the group positive for ERE and mean of 2366 for the group without disease) reported (Table 2). In terms of the relative abundance, in rabbits with ERE, there was obviously more Verrucomicrobia and Bacteroides, whilst in healthy animals, there was obviously more Firmicutes (Figure 4).

**Figure 5:**
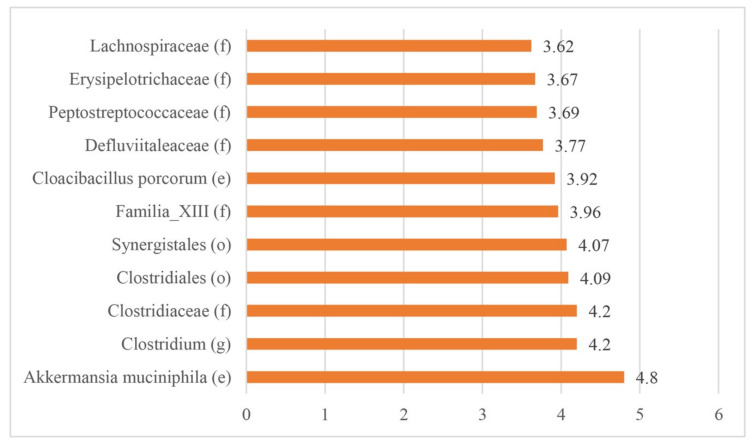
Biomarkers identified using the linear discriminant analysis effect size (LEfSe) test in rabbits with ERE. (f): family, (e): species, (o): order, (g): genus.

Following statistical analysis, ERE-positive rabbits showed a significantly higher abundance for some identifiable microorganisms. These gave an indication of organisms that could be used as biomarkers. Specifically, these were: *Akkermansia* (15.22% vs. 5.62%); *Clostridium* (3.56% vs. 0.61%); *Bacteroides* (2.67% vs. 0.92%); *Cloacibacillus* (1.72% vs. 0%); *Saccharimonas* (1.27% vs. 0.22%); *Synergistes* (0.71% vs. 0%); and *Erysipelatoclostridium* (0.49% vs. 0.03%). In the case of archea, *Methanosphaera* (0.58% vs. 0.09%) was the only genus that increased significantly. In addition, having the ERE disease significantly reduced the relative abundance of the genera *Subdoligranulum* (3.64% vs. 7.49%) and *Eisenbergiella* (0.03% vs. 1.27%) and the families Ruminococcaceae (26.34% vs. 34.12%) and Lachnospiraceae (8.14% vs. 23.49%) (Figure 5). 

#### 3.2.2. Bacterial Diversity

The diversity and richness of the microbiota of healthy rabbits was greater in all of the calculated indices compared to EEC-positive rabbits (Table 3).

#### 3.2.3. Biomarkers

**Figure 6:**
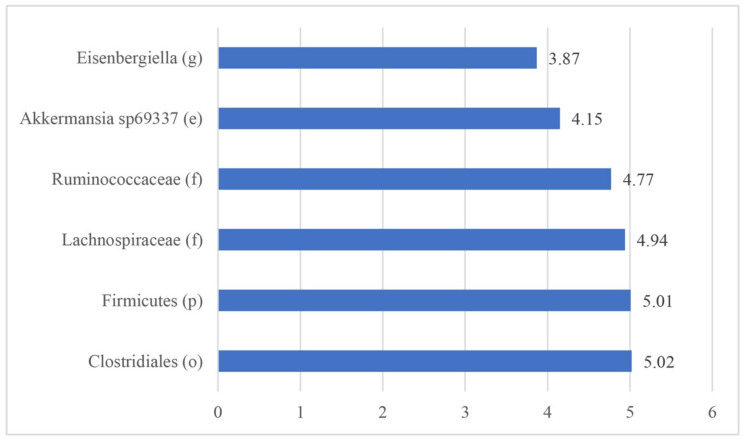
Biomarkers identified using the LEfSe test in healthy rabbits. (f): family, (e): species, (o): order, (g): genus, (p): phylum.

The LEfSe test reported 11 biomarkers in animals with ERE disease; however, 6 biomarkers were reported in healthy rabbits (Figure 5 and Figure 6).

## 4. Discussion

Macroscopic lesions observed were similar to those mentioned previously in several studies [4,9,11,28], giving us confidence that those assumed to be ERE positive at the outset were indeed suffering from ERE. Interestingly, however, we observed atrophy of the intestinal crypts in this study. This could be associated with two aspects caused by the syndrome: (1) The absence of food in the colon due to impaction of the cecum, and (2) the proliferation of microbial communities belonging to the family Clostridiaceae. In the case of microbial proliferation leading to digestive problems, this has been documented in broiler chickens [29] and children [30]. Cecal impaction promotes an environment for the proliferation of various pathogenic communities, and also causes food to not access the more distal sections of the gastrointestinal tract and so decreases the growth of resident communities, such as *Ruminococcus* spp. [31].

The mean number of sequences obtained during this analysis was around 14,500 for ERE+ animals and around 12,500 for ERE- animals. These values are slightly lower than some examples of previous works involving rabbits, e.g., mean of around 37,500 per sample [32] and 48,700 per sample [33]. However, they are still higher than similar works published on other herbivore systems, e.g., around 5500 per cow [34] and around 10,000 per deer [35]. However, the rarefaction curve showed good coverage and the debugging carried out by the DADA2 algorithm reinforces the accuracy of the identified OTUs and reduces bioinformatical error [21]. Comparison of the metataxonomic profiles showed a difference between groups (*p* < 0.04), which supported the classification of this enteropathy as dysbiosis. It has been reported that ERE dysbiosis affects a rabbit’s microbiota, because it increases the abundance of the phyla Bacteroidetes, Proteobacteria, and Verrucomicrobia [15,17]. However, it decreases the abundance of the phylum Firmicutes [15,17,18]. In addition, an effect on the diversity has been reported, principally on the genera *Butyricimonas* and *Anaerotruncus* [17].

In this study, an increase in abundance for the phyla Bacteroidetes, Saccharibacteria, Tenericutes, and Verrucomicrobia in ERE-infected animals was observed, but the phylum Firmicutes decreased. However, due to intra-group variation in the numbers, only Saccharibacteria and Tenericutes showed significant (*p* < 0.05) increases, with Verrucomicrobia showing a trend towards an increase (*p* = 0.068). Furthermore, the Synergistetes phylum was only found in ERE rabbits. However, the present study did not record the presence of *Escherichia–Shigella* or a significant change in the phylum Proteobacteria. Abecia et al. [17] already reported that the Synergistetes phylum has only been seen in rabbits with ERE; however, more recently it has been shown that it can also appear in healthy rabbits [18]. Furthermore, a recent study did not report the Synergistetes phylum in young rabbits [33]. In the case of *Escherichia–Shigella*, this genus has not been reported in some animals with ERE [17,33]; however, other studies showed that the genus can be present either in healthy or ERE rabbits [15,18].

The work presented here shows some differences in terms of the sequences detected relative to previous reports. Unlike many of the previous investigations into the microbiome of animals infected with ERE relative to healthy animals, we did not use a round of PCR prior to the sequencing stage, instead sequencing directly from the genomic DNA. This approach was used as potentially there have been differences between studies in the microbial profile, which may be related to the type of PCR amplification used prior to performing sequencing. For example, it has been shown that a prior round of PCR can present different forms of bias in terms of the sequences generated. The primers, which are described as being “universal”, are not always as universal as first described and may fail to amplify all intended targets [36,37]. In addition, although PCR is regarded as a reliable process, it is not error free and so amplification errors can in turn lead to sequencing errors down the line [36,37]. For this reason, some papers have suggested that, if possible, it may be prudent to forego any steps prior to sequencing, including initial rounds of PCR [38]. Moreover, in the case of PCR being performed prior to sequencing, this has been shown to have a distorting effect when using second-generation platforms, including affecting the calculations of diversity and relative abundance [39,40,41]. For the reasons given above, we decided that it was appropriate to consider a pre-sequencing round of PCR as an optional requirement. In doing so, we feel that the current data avoids issues associated with PCR bias and so should provide a more accurate reflection of the genomic DNA available for analysis at the point of DNA extraction. Given the fact that we managed to identify sequences from organisms not previously reported in the digestive tract of the rabbit, we feel this decision has been vindicated.

Other factors that could contribute to the differences relative to previous work could include the experimental diet, which can contribute to the stability of the phylum Proteobacteria. In the present study, the diet did not contain any type of antibiotic, another factor that was different from the work of Abecia et al. [17], where the diet offered contained antibiotics (bacitracin and robenidine). Other dietary variables between experiments also include the level of crude fiber (CF) and crude protein (CP), which are known to be important factors in terms of preventing, or reducing, the issue of ERE.

The increase in the abundance and decrease in diversity indices observed in this study corroborates that reported in some previous studies [15,17]. The Simpson index differs from that reported by Jin et al. [18], who did not detect a difference. This index does not discriminate between species with a low population and diversity is confirmed by observing other diversity indices, in which differences can be observed, as we showed in this study. In this study, the relative abundance of organisms in those with ERE was higher than in healthy animals, and the Chao1 index was 304.98. Previously, the ERE Chao1 index has been reported as 180.01 [17] and 191.4 [18]. However, the relative abundance in healthy rabbits was similar to those in earlier studies [17,18]. Differences in the values obtained in this study may be associated with the origin of the rabbits and the type of food offered; the rabbits were obtained from production farms and no type of antibiotic was offered in the diet unlike Abecia et al. [17], who had antibiotics in the diet, which may have an effect on the microbial profile [8,42]. In the case of Jin et al. [18], antibiotics were not offered, but rabbits were free of specific pathogens and were maintained under laboratory conditions, which could have a strong effect on the establishment of the colonization of microorganisms and can affect the diversity and abundance of the microbiota. 

Members of the phyla Verrucomicrobia (family Verrucomicrobiaceae), Firmicutes (families Clostridiaceae, Familia_XIII, Defluviitaleaceae, Peptostreptococcaceae, Erysipelotrichaceae, Lachnospiraceae), and Synergistetes (family Synergistaceae) were associated with the presence of ERE by LEfSe analysis (Figure 5). In addition, the following organisms were also found to be associated with ERE: *Akkermancia muciniphila*, *Clostridium* spp., and *Cloacibacillus porcorum*.

The Firmicutes phylum is the most abundant within the cecal microbiota of healthy rabbits, representing between 65% to 75% of the abundance [43,44,45]. In the present study, Firmicutes were the most abundant organisms in both groups, although the relative abundance was higher in healthy rabbits at 78.88%, relative to those with ERE with 59.26%, in keeping with previous studies [15,17,18].

We observed the Lachnospiraceae family in both healthy and ERE rabbits, in keeping with studies of Bäuerl et al. [15] and Abecia et al. [17], but unlike Jin et al. [18], who only found this family in healthy rabbits.

*Clostridium* spp. have been linked to ERE, primarily *C. perfringens* and *C. cuniculli* [7,31,46,47]. Djukovic et al. [31] identified some strains of *C. cuniculli* and proposed that in association with other *Clostridium* spp., they were involved in the development of ERE, although inoculation with them has not produced enteropathy. In this study, we found that *Clostridium* as a genus is associated with the presentation of ERE.

The phylum Verrucomicrobia, although present in healthy animals [43,44], has been implicated with the presence of ERE when it has been increased between 6% to 9.5% [15,18]. In the present study, the phylum Verrucomicrobia had an increase of 9.6% in ERE-infected animals and one species within the phylum was identified as an ERE biomarker: *Akkermansia muciniphila*. 

*Akkermansia muciniphila* has been associated with ERE previously [15,17,18], and it is known to degrade mucins and stimulate mucin production in the gastrointestinal tract [48]. Therefore, its presence can be related to the increase in mucus production in the small intestine and mucus accumulation in the cecum. Interestingly, human dysbiosis studies [49,50] showed that a low presence of this bacterium is associated with the presentation of ulcerative colitis, irritable bowel syndrome, and obesity. Moreover, the role that this microorganism plays in the preservation of the physical barrier of the intestine has been verified by stimulating the production of mucins [51,52,53]. However, the proliferation of this bacterium in ERE syndrome may be related either with the stimulation of globet cells or the interaction with other bacteria due to the disbalance of the bacterial population.

The Synesrgistetes phylum is a common inhabitant of the soil, as well as in anaerobic systems, such as the gastro-intestinal tract of pigs [19,54]. It has been associated with being an opportunistic pathogen in pigs [19] and an agent associated with oral dysbiosis in cattle [20]. Normally, they degrade mucin and show resistance to vancomycin [20]. *Cloacibacillus porcorum* belongs to the phylum Synergistetes and it can ferment amino acids and uses mucus as a carbon source [55]. In the present study, *C. porcorum* was identified only in ERE rabbits. Jin et al. [18] also reported *C. porcorum* in ERE rabbits but found it at a lower percentage in healthy rabbits. Some studies did not find any members of this phylum [5,17], while others [33,43,44] failed to identify this phylum in the cecal microbiota of healthy rabbits, and others [56] reported it in the hard feces of healthy rabbits. The presence of the genus may be associated with mucus production.

Metataxonomic studies can generate information that allows the theorizing and testing of specific treatment ideas for ERE. In this study, some bacteria were identified as potential ERE biomarkers, which appear to be strongly related with the disease. Recently, Read et al. [33] showed that cecal microbiota development starts from an early age under different feeding conditions (lactation and solid food), and the length of lactation time has a directly proportional effect with the cecal health of rabbits. Potentially poor handling during lactation and nursing can have a high impact on the cecal microbiota and can contribute to ERE syndrome development. Therefore, it is necessary to undertake some interaction experiments and more metataxonomic studies to elucidate the etiopathology of this syndrome and subsequently propose alternative treatments. The data presented here provides a new insight into candidate organisms that may be used as biomarkers to identify animals that may be susceptible to the development of ERE.

## 5. Conclusions

Rabbit epizootic enteropathy is a dysbiosis that presents a principally smaller size of the crypts in the colon. Microbial imbalance damages are related with the proliferation of communities belonging to the phyla Verrucomicrobia; (*Akkermansia muciniphila*), Firmicutes (*Clostridium* spp.), and Synergestes (e.g., *Cloacibacillus porcorum*). This is the first time that *Cloacibacillus porcorum* has been reported to have an association with ERE disease. 

## Figures and Tables

**Table 1:** Mean crypt depths in three regions of the rabbit intestine. Values shown are the mean of 10 crypts from the 6 animals per treatment; ERE+ (infected) versus ERE− (healthy).

Site	Depth of Crypts (µm)			
	ERE+	ERE−	SEM	*p* Value
Ileo-ceco-colic valve	199	187	16.21	0.73
Cecum	136	121	5.55	0.22
Colon	173	308	10.51	0.0004

**Table 2:** Mean abundance (n = 6 relative to health status) with standard error and percentage of the bacterial phyla of the cecal contents of rabbits with and without ERE. Where a phylum was not observed is denoted by “-“.

Phylum	ERE+	%	ERE−	%	*p* Value
Actinobacteria	538 ± 108.5	1.05	312 ± 217.9	0.73	0.138
Bacteroides	7327 ± 2934.8	14.32	2300 ± 1020.4	5.36	0.137
Cyanobacteria	286 ± 88.3	0.56	214 ± 58.1	0.50	0.508
Euryarchaeota	400 ± 97.3	0.78	387 ± 194.2	0.90	0.951
Firmicutes	30320 ± 4856.3	59.26	33848 ± 2811.8	78.88	0.544
Unidentified	3504 ± 1402.1	6.85	2366 ± 260.9	5.51	0.443
Proteobacteria	697 ± 225.8	1.36	521 ± 58.5	1.22	0.468
Saccharibacteria	683 ± 224.3	1.34	121 ± 95.3	0.28	0.044
Synergistetes	1287 ± 680.6	2.52	-	-	0.088
Tenericutes	39 ± 18.3	0.08	196 ± 47.3	0.46	0.012
Verrucomicrobia	6075 ± 1334.6	11.87	2642 ± 1012.8	6.16	0.068

**Table 3:** Summary of sequences identified and comparison of microbial diversity indices in rabbit cecal contents of animals with and without ERE. ERE+: Rabbits positive for epizootic enteropathy. ERE−: Healthy rabbits.

Sequences and Index.	ERE+	ERE−	*p*-Value
Number of sequences	115,534	99,863	
Number of operational taxonomic units (OTU)	317	279	
Number of identified genera	76	81	
Shannon Index	6.24 (0.18)	6.54 (0.04)	0.001
Simpson Index	0.95 (0.04)	0.97 (0.01)	0.001
Simpson Inverse Index	30.12 (0.09)	43.54 (0.06)	0.001
Fisher Index	66.20 (2.19)	81.40 (0.80)	0.001
Chao1 Index	304.98 (6.70)	275.86 (2.50)	0.002

## Supplementary Materials

The following are available online at https://www.mdpi.com/2076-2615/10/6/936/s1, Table S1; title: Relative abundance of the Phyla, Classes, Orders, Families and Genera in the caecal and faecal samples. Values with an abundance value of less than 0.001 are denoted as “low”.
